# A Novel STAT3 Gene Mutation Related Hyper-IgE Syndrome Misdiagnosed as Hidradenitis Suppurativa

**DOI:** 10.1155/2018/4860902

**Published:** 2018-08-13

**Authors:** Pragya Shrestha, Geetika Sabharwal, Gisoo Ghaffari

**Affiliations:** ^1^Internal Medicine Department, Reading Hospital-Tower Health System, Reading, PA, USA; ^2^Division of Pulmonary, Allergy, and Critical Care Medicine, Penn State College of Medicine, Hershey, PA, USA

## Abstract

Although Hyper-IgE Syndrome (HIES) is a rare immunodeficiency disorder, presenting symptoms may be as common as lung and skin infections. Symptoms are usually nonspecific such as recurrent abscesses, folliculitis, and pneumonias along with skeletal abnormalities. Careful history of susceptibility to skin and lung infections, thorough family history, and findings on physical exam can guide towards the diagnosis of this often-eluded condition. Early optimization of therapy with prophylactic antibiotics can prevent recurrent infections and future complications and improve quality of life and longevity of survival. We present a case of a young female with Hyper-IgE Syndrome with a novel mutation in STAT 3 gene who initially presented with long standing history of intractable skin abscesses being managed as Hidradenitis Suppurativa.

## 1. Background

Hyper-IgE syndrome (HIES), also commonly known as Job's syndrome, was first described by Davis et al. in 1966 as a syndrome associated with severe dermatitis with “cold” abscesses [1, 2]. Subsequently, this was further characterized by typical facial appearance with high levels of immunoglobulin E (IgE) by Buckley et al. in 1972 [3]. Since then, additional features of HIES have been recognized and it has since been regarded as a multisystem disorder characterized by chronic eczema, recurrent staphylococcal skin and lungs infections, pneumatocele formation, candidiasis, retained primary teeth, joint hyperextensibility, low bone density with bone fractures, scoliosis, and craniosynostosis [4, 5].

It is a rare immunological disorder with an estimated incidence of 1 in 500,000 to 100,000 individuals [4, 6] with both autosomal dominant (AD) and recessive forms (AR) of inheritance [1, 7]. Mutation in the signal transduction and activation of transcription 3 gene (STAT3) with gain-of-function is the most prevalent mutation described and accounts for majority of its autosomal dominant and sporadic forms [1, 5, 7]. Tyrosine kinase 2 (TYK2) and dedicator of cytokinesis 8 (DOCK8) genes mutations are implicated in recessive forms [7, 8]. Unlike AD-HIES, they have higher predisposition to viral infections, severe atopic eczema, food allergy, neurologic symptoms, and malignancies. [9]

National Institute of Health (NIH) clinical HIES scoring system was developed in 1999 based on 19 clinical and laboratory findings [4]. This gave an estimate of likelihood of HIES diagnosis with more than 40 points suggestive of the diagnosis [4, 8]. Genetic study for mutation analysis is the mainstay of diagnosis confirmation. Among the STAT3 gene mutations in literature for AD-HIES, V463E variant of STAT3 gene with a “loss of function” (located at the DNA-binding domain), found in our patient, has neither been published as a pathogenic nor been a benign variant to our knowledge. The variant was not observed in large population cohorts [10].

## 2. Case Presentation

A 21-year-old white female was referred to our Allergy-Immunology Clinic for a history of multiple intractable cutaneous abscesses and cysts for several years. She had undergone multiple incision and drainage and had been treated with antibiotics as well as topical and systemic steroids intermittently with minimal relief and developed methicillin resistant staphylococcal aureus (MRSA) colonization during the same period. She was at the time clinically diagnosed as Hidradenitis Suppurativa. Her medical history was significant for hypertension, diabetes mellitus type II, hyperlipidemia, obesity, and anxiety.

On further questioning, she reported more than fifteen hospitalizations for pneumonias. According to her mother, she had recurrent pneumonias, upper respiratory tract infections, sinusitis, mastoiditis, and oral candidiasis since early childhood. The patient denied any history of atopic dermatitis, other types of eczema, or food allergy. Evaluation for cystic fibrosis and hypogammaglobulinemia at the time had been negative. More recently, she had been admitted for septic shock secondary to septic arthritis of left hip. She also had multiple fractures with minimal trauma since childhood and was clinically diagnosed with osteogenesis imperfecta. She also reported history of primary teeth retention for which she had underwent orthodontic surgery at the age of 12 years. She had a normal birth history with normal documented developmental milestones and was up-to-date with her immunizations. She had Penicillin and Trimethoprim-Sulfamethoxazole (TMP-SMX) listed as allergy after she developed rash with their use when she was a toddler.

Family history was pertinent for her paternal grandmother with recurrent pneumonias, who passed away at 40 years of age secondary to a severe lung infection. Her biological brother had a history of recurrent skin boils.

Important physical findings included coarse facial feature with exacerbated pore size, deep set eyes, broad nasal bridge, high arched palate, and multiple scattered healed scars on skin with some remnant cold abscesses. She had mild thoracic scoliosis. Pulmonary and cardiovascular exam were unremarkable.

Laboratory investigations revealed a normal complete blood count with no eosinophilia. IgE level was elevated at 5,842 IU/ml, with erythrocyte sedimentation rate of 64 mm/hr and C-reactive protein of 1.56 mg/dl. IgG, IgM, and IgA levels were within normal limit. Tetanus and pneumonia titers were normal. Total complement (CH50) levels were normal and testing for chronic granulomatous disease was unrevealing. Due to history, physical findings, and these initial labs, we suspected Hyper-IgE Syndrome. A calculated HIES score was 63 (>40 required for diagnosis). With a high suspicion of AD-HIES, mutation analysis of STAT3 gene was sent which detected a novel pathogenic mutation, C.1388 T>A (pVal463Glu) at DNA-binding domain of STAT3 gene. Although this mutation in STAT3 gene has never been published as a pathogenic mutation leading to AD-HIES, missense variants in nearby residues have been reported in association to those reported in the Human Gene Mutation Database in association with HIES [11].

Penicillin allergy testing and TMP-SMX oral challenge were performed in the clinic. With a normal response, she was started on TMP-SMX (800mg-160mg) once daily prophylactically for prevention of infections (MRSA sensitive to TMP-SMX).

Patient was advised to follow up for clinical response and monitoring tolerance to treatment. She was asked to have regular dental exam, chest imaging, bone density scan, and pulmonary function test screening. She was also advised to have genetic counselling. Genetic testing for her biological brother was offered.

## 3. Discussion

Mutations in STAT3 gene account for majority of autosomal and sporadic HIES [1, 5, 7]. Located in human chromosome 17q21, STAT3 plays a vital role in signal transduction induced by many cytokines (IL-6, IL-10, IL-17, IL-21, IL-22) [4, 12]. Its mutation yields abnormal inflammatory response leading to immunological abnormalities and higher predisposition to infections [4]. Our patient had pathogenic mutation of V463E, a nonconservative amino acid substitution. This variant is located within the DNA-binding domain, and missense variants in nearby residues have been reported [11]. Pathogenic variants described to date include missense, single amino acid in-frame deletions, and splice variants, all being “gain-of-function” related mutations. Several hotspot variants in SH2 and DNA-binding domain are known with four recurrent variants occurring at CpG dinucleotides: c.1144C>T, c.1145G>A, c.1268G>A, and c.1909G>A as well as a recurrent three-base in-frame deletion c.1387_1389delGTG [13].

**Figure 1:**
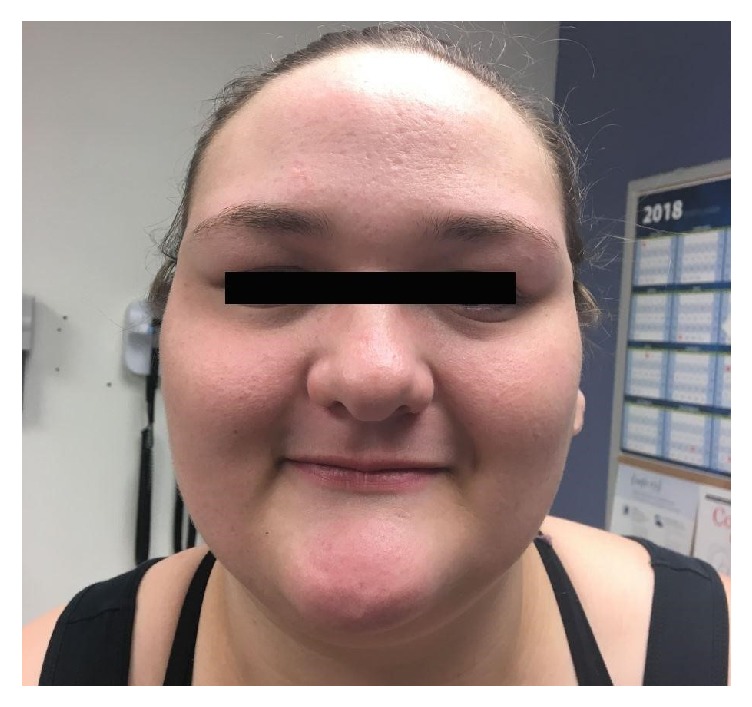
Facial characteristic of AD-HIES patient: coarse facial feature with exacerbated pore size, deep set eyes, and broad nasal bridge.

**Figure 2:**
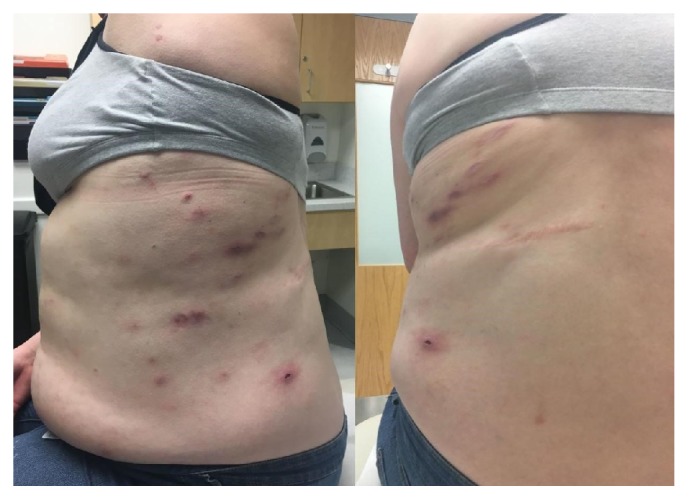
Scattered healed scars on skin with some remnant cold abscesses.

**Figure 3:**
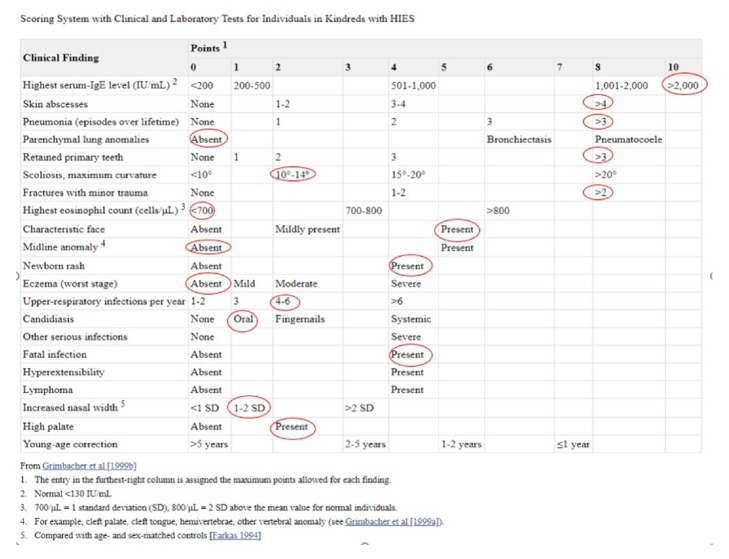
Hyper-IgE Syndrome/Grimbacher scale: our patient score highlighted (total score: 63).

This entity is a rare disease without any known prevalence and does not have any preference for sex or race [3]. The disease severity also does not correlate with the individual's IgE level. [14] The criteria of HIES or Grimbacher scale provide insight into times of diagnostic dilemma (Figure 3); however, definitive test relies on genetic and molecular analysis [1, 8]. In the case described, high index of suspicion was made due to presence of cutaneous abscesses (Figure 2), colonization with MRSA, high level of IgE, recurrent pneumonia, history of retained primary teeth, multiple fractures with minimal trauma, scoliosis, and typical facial characteristics (Figure 1).

Long-term antibiotic therapy with antistaphylococcal activity (e.g., TMP-SMX, Penicillin, Cephalosporins) can contribute to significant reduction in skin and lung infections such that risk of developing serious future infections, pneumatoceles, and lung damage outweighs the risk of developing antibiotic resistance [4, 8, 15]. Among immunomodulatory agents, subcutaneous Interferon-gamma (0.05mg/m^2^ three times weekly) has been shown to decrease IgE levels and infection susceptibility; however, no randomized control trials exist [13]. Recently, a monoclonal anti-IgE, omalizumab has been shown to help decline serum IgE with improvement in atopic conditions; however, in Hyper-IgE state, this benefit is unknown and an area of further study [13, 15].

## 4. Learning Objectives

- Identification of Hyper-IgE Syndrome in patients with characteristic history and clinical features can assist in early diagnosis and help initiate preventive measures for future infections and complications.
- Clinical features can determine the type of HIES; confirmatory test is genetic testing and molecular analysis.
- HIES can be a sporadic or hereditary disorder. V463E variant of STAT3 gene, located at the DNA-binding domain with loss of function mutation, is a novel mutation seen in our patient that could be associated with this disease entity.
